# Lack of association of *FKBP5* SNPs and haplotypes with susceptibility and treatment response phenotypes in Han Chinese with major depressive disorder: A pilot case–control study (STROBE)

**DOI:** 10.1097/MD.0000000000026983

**Published:** 2021-09-10

**Authors:** Chenghao Yang, Shen Li, Yanyan Ma, Bing Chen, Meijuan Li, Fokko J. Bosker, Jie Li, Ilja M. Nolte

**Affiliations:** a Biological psychiatry Laboratory, Tianjin Mental Health Institute, Tianjin Anding Hospital, Tianjin, China; b University of Groningen, University Medical Centre Groningen, University Centre of Psychiatry, Groningen, the Netherlands; c Department of Psychiatry, College of Basic Medical Sciences, Tianjin Medical University, Tianjin, China; d University of Groningen, Research School Behavioral and Cognitive Neurosciences (BCN); e University of Groningen, University Medical Center Groningen, Department of Epidemiology, Groningen, The Netherlands.

**Keywords:** antidepressant treatment resistance, *FKBP5*, major depressive disorder, single nucleotide polymorphism, susceptibility

## Abstract

The identification of single-nucleotide polymorphisms (SNPs) in genes putatively related to pathophysiological processes in major depressive disorder (MDD) might improve both diagnosis and personalized treatment strategies eventually leading to more effective interventions. Considering the important role of the glucocorticoid receptor and the related FK506 binding protein 51 (FKBP51) in the pathophysiology of MDD, we aimed to investigate putative associations between variants of *FKBP5*, the coding gene of FKBP51, with antidepressant treatment resistance and MDD susceptibility.

Nine common SNPs of the *FKBP5* gene prioritized based on location and, putative or known functions were genotyped in Han Chinese population, including MDD patients with or without antidepressant-treatment resistance and healthy controls. Associations of *FKBP5* SNPs with MDD susceptibility and treatment response were examined in the whole group of MDD patients, as well as in subgroups stratified by antidepressant treatment resistance, compared with healthy controls.

In total, 181 Han Chinese patients with MDD and 80 healthy controls were recruited. No significant SNP or haplotype associations were observed in the whole patient group. There were nominal significant differences both for the haplotype block with SNPs in strong LD (*r*^2^ > 0.8, *P* = .040) and haplotype block with SNPs in moderate LD (*r*^2^ > 0.1, *P* = .017) between the haplotype distributions of patients with antidepressant treatment resistance (n = 81) and healthy controls, but both significances did not survive multiple testing correction. Furthermore, no specific haplotype could be observed causing a significant difference in any combination between all comparisons.

No associations were observed of *FKBP5* variants with MDD or antidepressant treatment response. The lack of associations might be due to the relatively small sample size of this study (power ranged from 0.100 to 0.752). A follow-up study will need larger, better phenotyped, and more homogeneous samples to draw a definitive conclusion regarding the involvement of this gene in MDD.

## 1 Introduction

Major depressive disorder (MDD) is a widespread mental illness that affects approximately 350 million people worldwide.^[1]^ The disease burden caused by depression accounted for 4.3% of the total disability-adjusted life years in 2004 and MDD is expected to become the leading cause of reduced quality of life by the year 2030.^[1]^ Moreover, lifetime suicide risk for MDD is 2.2% to 15% making the development of more effective treatment strategies an urgent matter.^[2]^ Indeed 30% to 50% of the MDD patients do not respond satisfactorily to antidepressant drugs even after sufficient forms of treatment, eventually falling in the category of treatment-resistant depression.^[3]^

The considerable individual variation in antidepressant treatment response attracted a lot of attention for the underlying mechanisms of treatment resistance. Earlier studies have suggested that a dysregulated inflammatory system is involved in the pathophysiology of MDD,^[4]^ and that this may also contribute to antidepressant treatment resistance.^[5]^ Furthermore, a dysregulated hypothalamic-pituitary-adrenal (HPA) axis is a key characteristic of depression, which can be endorsed by inflammatory cytokines.^[6,7]^ In this regard, a dysfunction of HPA axis could also be an important factor with antidepressant treatment resistance. The FK506-binding protein 51 (FKBP51) is a chaperon protein supporting glucocorticoid receptor (GR) maturation^[8]^ and thus plays an important role in regulating GR activity.^[9]^ Under physiological conditions FKBP51 is part of a negative feedback loop attenuating stress-induced increases of plasma cortisol^[10]^; however, FKBP51 hyperactivity may result in an increased GR resistance.^[11]^ It has also been shown that FKBP51 can work as a scaffolding protein leading to the dephosphorylation of Akt.^[12]^ This decrease of Akt signaling activity may result in decreased neurogenesis,^[13]^ which might also be involved in treatment resistance.^[14,15]^ In addition, FKBP51 has been reported to reduce inflammatory responses through a reduction of transcriptional factor NF-κB (p50/p65) nucleus translocation.^[16]^

The identification of single-nucleotide polymorphisms (SNPs) relating to treatment response could help to understand the varying responses to antidepressant treatment and aid the development of better treatment strategies. Several lines of evidence have shown that SNPs in *FKBP5*, the gene encoding the FKBP51 protein, may be involved in the pathophysiology of MDD. For instance, a meta-analysis showed that the SNPs rs1360780 and rs3800373 in the *FKBP5* gene increased the risk of developing MDD and were also associated with the risk of suicidal behavior in whites.^[17]^ Furthermore, a case–control study in 202 Korean demonstrated that the T allele of *FKBP5* rs1360780 was associated with significant volume reductions in mood-related cortical and subcortical regions in MDD patients compared to controls.^[18]^ In addition, a study within STAR∗D demonstrated that rs352428 significantly decreased the gene's transcriptional activity resulting in reduced protein expression, which was significantly associated with an insufficient response to selective serotonin reuptake inhibitors.^[19]^ So for the relation between *FKBP5* polymorphisms and antidepressant treatment response has only be studied in Caucasians and it remains unclear whether the observed effects can be generalized to other ethnicities. Finally, Cattaneo et al reported a 11% reduction in leukocyte FKBP51 RNA expression in MDD patients responding to 8-week antidepressant treatment (citalopram or nortriptyline), whereas no such change was found in nonresponders.^[20]^ Because of its key role in glucocorticoid pathways, neurogenesis, and inflammation. FKBP51 is a promising target to investigate the underlying mechanisms of antidepressant treatment resistance.

Technological innovations like genome-wide association studies (GWAS) have substantially aided in the discovery of genetic risk factors for (partially) hereditary diseases.^[21]^ However, thus far GWAS of depression were far less successful, which may be partly attributed to the heterogeneous study population collected from many different sites to increase sample size,^[22–24]^ based on the current diagnostic classification.^[25]^ The CONVERGE study detected two loci for MDD in 11,670 Han Chinese with only a tenth of the estimated sample size, through increasing homogeneity of studying population by stringent criteria and deep phenotyping,^[22]^ which would indicate that population homogeneity is critical for the detection of genetic associations of MDD.^[26]^ Therefore, in this study, we focused on a homogeneous population of Han Chinese patients with antidepressant treatment resistance and increased inflammatory activity (we will refer to this group of patients as TRDI in the following text). We compared this subgroup of patients with Han Chinese MDD patients without treatment resistance (MDNTR in the following text) and healthy controls (all from Tianjin, China). The aim of this study was to investigate the role of *FKBP5* genetic polymorphisms in MDD vulnerability and antidepressant treatment response. In this explorative study, we hypothesized that *FKBP5* polymorphisms, including allele, genotype and haplotype distributions, are contributable to increased MDD susceptibility and antidepressant treatment resistance in Han Chinese population, particularly in TRDI patients.

## 2 Materials and methods

### 2.1 Participants and design

This study recruited 3 groups of participants, including TRDI patients, MDNTR patients, and healthy controls. The TRDI patients (n = 81) were recruited from the inpatient and outpatient departments of Tianjin Anding Hospital between September 2015 and October 2018, and took part in the clinical study registered on “ClinicalTrials.gov” with protocol ID “NAC-2015-TJAH” and ClinicalTrials.gov ID “NCT02972398”. The TRDI patients were called back for blood sampling. Inclusion criteria were: a current episode of MDD diagnosed according to Diagnostic and Statistical Manual of Mental Disorders, Fourth Edition, Text Revision (DSM-IV-TR) with Structured Clinical Interview for DSM-IV; age between 18 and 65 years; a total score of 17 items Hamilton Depression Rating Scale (HAMD-17) ≥17; a C-reactive protein level between 0.85 and 10 mg/L; insufficient response to ≥1 antidepressants given for at least 6 weeks and in an adequate dose during the current episode. Exclusion criteria were: a history of manic episode; use of mood stabilizer; use of antipsychotic medication with more than half of the maximum dosage suggested in the instruction; history of substance abuse or dependence; an allergic reaction to NAC or any component of the preparation; severe somatic diseases that might interfere with regular antidepressant treatment including conditions such as kidney and liver failure, uncontrolled hypertension, cardiovascular, cerebrovascular and pulmonary disease, thyroid disease, diabetes, epilepsy and asthma; use of anti-inflammatory medication for >7 days in the last 2 months preceding the trial; use of immunosuppressive medication such as oral steroid hormones; history of chronic infection, such as tuberculosis, AIDS, hepatitis; C-reactive protein value >10 mg/L; women in pregnancy or lactation period. More detailed information on study procedures has been described elsewhere.^[27]^

**Figure 1:**
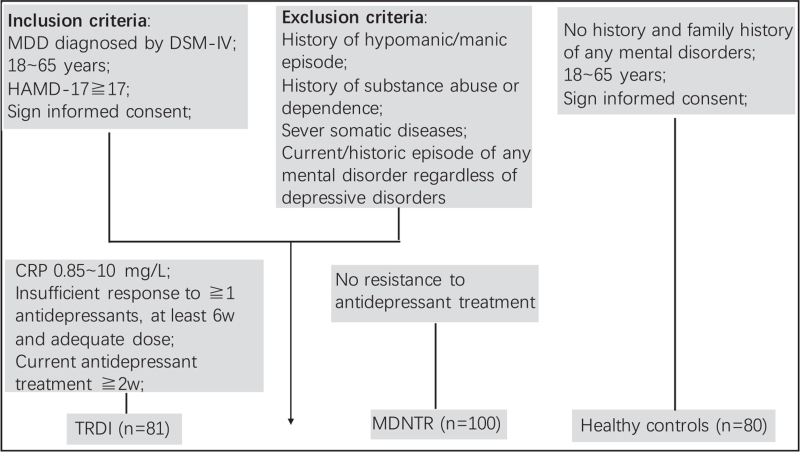
Flowchart of recruitment. CRP = C-reactive protein, DSM-IV-TR = Diagnostic and Statistical Manual of Mental Disorders, Fourth Edition, Text Revision, HAMD-17 = 17 items Hamilton Depression Rating Scale, MDD = major depressive disorder, MDNTR = major depression without treatment resistance, TRDI = antidepressant treatment resistance and increased inflammatory activity.

The data of MDNTR patients (n = 100) and healthy controls (n = 80) came from the inpatient and outpatient departments of Tianjin Anding Hospital and Tianjin General Hospital during November 2009 and July 2010. The inclusion criteria for MDNTR patients were: diagnosed MDD with DSM-IV, first episode or recurrent; no resistance to anti-depressant treatments, that is, defined the present episode as a relapse from the efficacious antidepressant treatment because of drug withdrawal for first-episode patients or a recurrence with a history of effective antidepressant treatments for recurrent patients; no history of manic or hypomanic episodes; total score of HAMD-17 ≥17. Patients were excluded if the current depressive disorder was not idiopathic but secondary to other conditions, like substance abuse, medical diseases, and so on; current or historic episode of any mental disorder regardless of depressive disorders; women in menstruation, pregnancy, or lactation period. To be clarified, the MDNTR patients were not assessed for inflammatory activity. The healthy controls had to have no history or family history of any mental disorders and no medical diseases. Medical diseases included severe somatic diseases such as kidney and liver failure, uncontrolled hypertension, cardiovascular, cerebrovascular and pulmonary disease, thyroid disease, diabetes, epilepsy and asthma. Both studies were evaluated by the Medical Ethical Board of the Tianjin Anding Hospital (Register number: tjad2015001 and tjad2009003, respectively) and all patients provided written informed consent. Please see the Figure 1 for flowchart of recruiting participants of this study.

### 2.2 Candidate SNPs selection

Nine SNPs of the *FKBP5* gene were prioritized for location and, putative or known functions, based on NCBI dbSNP and earlier reports on their associations with clinical phenotypes.^[28–30]^ Furthermore, the SNPs were considered for those above 15% in Han Chinese population according to minor allele frequency (MAF: 0.15∼0.26). These SNPs included rs1043805 (Chr6.35573655, 3’UTR, MAF 0.16), rs3800373 (Chr6.35574699, 3’UTR, MAF 0.21), rs9296158 (Chr6.35599305, intronic region, MAF 0.22), rs7748266 (Chr6.35624967, intronic region, MAF 0.15), rs1360780 (Chr6.35639794, intronic region, MAF 0.21), rs2766537 (Chr6.35729109, promotor region, MAF 0.35), rs9394309 (Chr6.35654004, intronic region, MAF 0.19), rs9470080 (Chr6.35678658, intronic region, MAF 0.26), and rs2817035 (Chr6.35728586, promotor region, MAF 0.19), and the chromosome positions are based on hg38.

### 2.3 Genotyping and quality control

Genomic DNA was extracted from 5 mL venous blood sample using the high-salt method,^[31]^ which was stored and processed at the Tianjin Anding Hospital or the Molecular or Population Genetic Center of Tianjin Medical University. For MDNTR patients and healthy controls, their samples had been storing at minus 80°C, which were unfreezed in 4°C refrigerator before genotyping. Genotyping was performed by matrix-assisted laser desorption time-of-flight mass spectrometry, which is a high-throughput blood group genotyping method. After inputting to the AssayDesigner3.1 software for the design of the target SNPs, primers were confirmed and synthesized. Polymerase chain reaction amplification, Shrimp Alkaline Phosphatase purification reaction, extension reaction, resin purification and detection on sequenom were performed on the Sequenom MassArrays platform. Ten percent of samples were used for re-genotyping randomly for quality control, to check the concordance rate. Genotype calling was done blinded to the participants’ clinical data.

The quality of the SNPs was checked by determining the call rate and the Hardy-Weinberg equilibrium (HWE) *P* value. SNPs were excluded if the call rate was <90% or the HWE *P* value among the healthy controls was <.05/9 = 5.5 × 10^–3^.

### 2.4 Statistical analysis

The allele and genotype frequency, call rate, HWE, and odds ratios were evaluated using PLINK v1.9. The *χ*^2^ test was used to compare the genotype frequency between cases (TRDI patient and MDNTR patient groups) versus healthy controls, TRDI versus healthy controls, MDNTR versus healthy controls, and also stratified patient groups by treatment response phenotype (treatment-resistant depression vs MDNTR). Analyses correcting for age and sex were performed using logistic regression with covariates. To define haplotype blocks, PLINK v1.9 was used to determine linkage disequilibrium (LD) between markers within 1Mb. For each chromosomal region haplotype blocks were next constructed using a lenient *r*^2^ threshold of 0.1 and using a stringent *r*^2^ threshold of 0.8. Haplotype frequencies within each haplotype block were then determined for cases and controls separately and compared using a permutation test as implemented in PHASE 2.1.1.^[32]^ In this permutation test case–control status was permuted over the individuals 10,000 times and the *P* value was determined as the proportion of tests from the permuted data with a *P* value smaller than that when using the original case and control datasets.

To avoid false-positive findings upon the multiple testing, a multiple testing correction was applied. Spectral decomposition of the genotype data was used to determine the number of independent test.^[33]^ The significance threshold in this study was 2.5 × 10^–3^, (=0.05/[5 {independent SNPs} × 4 {subgroup analyses}]).

The power analysis was performed using “Genetic Power Calculator” online (http://zzz.bwh.harvard.edu/gpc/cc2.html)

## 3 Results

### 3.1 Participant demographics and characteristics

A total of 261 Han Chinese participants was recruited, including a TRDI patient group (n = 81), a MDNTR patient group (n = 100), and a healthy control group (n = 80). The distributions of age and sex in the 3 groups are significantly different (TRDI vs HC, 46.0 ± 12.7 vs 40.5 ± 11.6, *P* = .003) and sex (*χ*^2^ = 23.8, *P* < .001). See Table 1 for details.

**Table 1:** Demographics and characteristics of participants.

		Age		Sex		
Characteristic	No.	Mean (SD)	*P*	Male (%)	*χ* ^2^	*P*
TRDI group	81	46.0 (12.7)	—	47 (58.0)	—	—
MDNTR group	100	42.8 (10.2)	—	24 (24.0)	—	—
HC group	80	40.5 (11.6)	—	25 (31.3)	—	—
TRDI vs MDNTR	—	—	0.062	—		<.001
TRDI vs HC	—	–	0.003	—	23.798	
MDNTR vs HC	—	—	0.183	—		

### 3.2 Individual SNP association study

#### 3.2.1 Case–control analysis

SNP rs2766537 was excluded from the analysis due to a too low call rate (49%). The SNP rs9394309 was not in HWE in the healthy controls (*P* = 3.7 × 10^–3^). Comparing the allele or genotype frequencies of cases and controls, we found no significant associations between any of the alleles or genotypes with MDD (Table 2). There were also no significant associations observed when comparing allele and genotype frequencies between MDNTR patients and healthy controls and between TRDI patients and healthy controls. Adjustment for sex and age did not change these results.

**Table 2:** Associations of genotype and allele of *FKBP5* SNPs between cases with major depressive disorder and controls.

		Genotype (subject size)		Allele frequency (%)				
SNP	Genotypes	Cases (n = 181)	HC (n = 80)	Cases	HC	*χ* ^2^	OR (95% CI)	*P*/*P* adjusted
rs1043805	TT/TA/AA	9/61/111	2/21/57	T 21.7	15.6	2.53	1.50 (0.91–2.49)	.12/.10
rs3800373	CC/CA/AA	12/69/100	3/24/53	C 25.6	18.8	2.70	1.48 (0.93–2.37)	.11/.12
rs9296158	AA/AG/GG	18/78/85	7/33/40	A 31.6	28.8	0.42	1.15 (0.76–1.73)	.54/.56
rs7748266	TT/TC/CC	6/53/122	2/20/58	T 17.7	15.1	0.51	1.21 (0.72–2.04)	.52/.48
rs1360780	TT/TC/CC	11/67/103	3/25/52	T 24.7	19.0	2.02	1.40 (0.88–2.23)	.17/.15
rs2817035	AA/AG/GG	6/55/120	3/26/51	A 18.6	20.0	0.13	0.92 (0.57–1.47)	.72/.66
rs9470080	TT/TC/CC	13/71/97	5/29/46	T 26.5	24.0	0.34	1.14 (0.73–1.79)	.58/.73

		TRDI (n = 81)	HC (n = 80)	TRDI	HC			
rs1043805	TT/TA/AA	4/29/48	2/21/57	T 23.4	15.6	3.04	1.66 (0.94–2.93)	.088/.063
rs3800373	CC/CA/AA	6/32/43	3/24/53	C 27.6	18.8	3.31	1.64 (0.96–2.80)	.081/.079
rs9296158	AA/AG/GG	9/36/36	7/33/40	A 33.1	28.8	0.70	1.23 (0.76–1.98)	.46/.39
rs7748266	TT/TC/CC	2/24/55	2/20/58	T 17.8	15.1	0.38	1.21 (0.66–2.23)	.64/.61
rs1360780	TT/TC/CC	4/28/49	3/25/52	T 22.4	19.0	0.57	1.23 (0.71–2.13)	.49/.40
rs2817035	AA/AG/GG	3/25/53	3/26/51	A 18.8	20.0	0.07	0.93 (0.53–1.62)	.89/.85
rs9470080	TT/TC/CC	7/33/41	5/29/46	T 28.9	24.0	0.89	1.29 (0.76–2.17)	.36/.53

		MDNTR (n = 100)	HC (n = 80)	MDNTR	HC			
rs1043805	TT/TA/AA	4/32/64	2/21/57	T 20.3	15.6	1.28	1.38 (0.79–2.42)	.27/.16
rs3800373	CC/CA/AA	6/36/58	3/24/53	C 24.0	18.8	1.32	1.36 (0.81–2.29)	.29/.18
rs9296158	AA/AG/GG	9/42/49	7/33/40	A 30.4	28.8	0.12	1.08 (0.68–1.71)	.82/.59
rs7748266	TT/TC/CC	3/29/68	2/20/58	T 17.7	15.1	0.41	1.21 (0.68–2.15)	.56/.38
rs1360780	TT/TC/CC	7/39/54	3/25/52	T 26.6	19.0	2.80	1.54 (0.93–2.57)	.10/.074
rs2817035	AA/AG/GG	4/30/66	3/26/51	A 18.5	20.0	0.13	0.91 (0.54–1.54)	.79/.75
rs9470080	TT/TC/CC	6/37/57	5/29/46	T 24.7	24.0	0.02	1.04 (0.63–1.72)	.90/.77

#### 3.2.2 Antidepressant treatment response analysis

The results of the genetic association analysis for treatment response are shown in Table 3. When comparing allele and genotype frequencies between MDNTR and TRDI patients, we did not find any significant differences in distributions of alleles and genotypes.

**Table 3:** Associations of allele and genotype between *FKBP5* SNPs and treatment response.

		Genotype (subject size)		Allele frequency (%)				
SNP	Genotypes	MDNTR (n = 100)	TRDI (n = 81)	MDN	TR TRDI	*χ* ^2^	OR (95% CI)	*P*/*P* adjusted
rs1043805	TT/TA/AA	4/32/64	4/29/48	T 20.3	23.4	0.49	1.20 (0.72–2.00)	.52/.52
rs3800373	CC/CA/AA	6/36/58	6/32/43	C 24.0	27.6	0.59	1.21 (0.75–1.96)	.46/.68
rs9296158	AA/AG/GG	9/42/49	9/36/36	A 30.4	33.1	0.29	1.13 (0.72–1.78)	.64/.76
rs7748266	TT/TC/CC	3/29/68	2/24/55	T 17.7	17.8	0.000017	1.00 0.58–1.75)	1.00/.97
rs1360780	TT/TC/CC	7/39/54	4/28/49	T 26.6	22.4	0.79	0.80 (0.49–1.31)	.39/.47
rs2817035	AA/AG/GG	4/30/66	3/25/53	A 18.5	18.8	0.01	1.02 (0.60–1.75)	1.00/.94
rs9470080	TT/TC/CC	6/37/57	7/33/41	T 24.7	28.9	0.71	1.24 (0.75–2.02)	.45/.62

### 3.3 Haplotype association study

We studied 2 haplotype blocks: one including SNPs rs1043805, rs3800373, rs9296158, rs7748266, rs136078, rs9470080, and rs2817035, which were all in at least moderate LD with a lenient *r*^2^ threshold of 0.1, and the other including only SNPs rs3800373 and rs1360780 that were in strong LD with each other (*r*^2^ > 0.8). We tested each haplotype for frequency differences in relation to depression and treatment response.

#### 3.3.1 Case-control analysis

The results demonstrated that there were no statistical differences in haplotype distribution between cases and healthy controls neither for the haplotype with the lenient nor for the one with the stringent *r*^2^ threshold (*P* = .26/.12, respectively), and also not between MDNTR and healthy controls (*P* = .59/.61). When comparing the haplotype distribution between TRDI patients and healthy controls, there were nominal significant differences both in the stringent *r*^2^ threshold haplotype block (*P* = .04) and the lenient *r*^2^ threshold haplotype block (*P* = .0147). However, both significances did not survive multiple testing correction. Furthermore, no specific haplotypes revealed significant differences in any of the analyses. The details of the haplotype analyses comparing cases to controls are shown in Table 4.

**Table 4:** Associations of haplotypes of *FKBP5* SNPs between MDD cases (TRDI/MDNTR) and controls.

	HC	Cases			MDNTR			TRDI		
Haplotype combination	Frequency%	Frequency%	OR	*P*	Frequency%	OR	*P*	Frequency%	OR	*P*
Haplotype block 1 (lenient *r*^2^ threshold)										
A-A-G-C-C-C-G	64.4	60.5	0.85	.55	61.3	0.87	.66	58.5	0.78	.41
T-C-A-T-T-T-A	8.4	7.3	0.87	.77	8.6	1.10	.87	5.9	0.69	.52
A-A-A-C-C-T-G	4.5	3.9	0.86	.82	3.7	0.84	.82	5.4	1.19	.80
A-A-G-C-C-C-A	3.6	1.8	0.49	.39	1.9	0.50	.46	1.2	0.38	.41
T-C-A-T-T-T-G	3.3	4.7	1.46	.60	4.2	1.27	.76	5.2	1.62	.52
A-A-A-C-C-T-A	3.1	1.6	0.51	.44	1.3	0.51	.55	1.0	0.41	.48
A-A-A-C-C-C-G	2.6	1.6	0.59	.56	2.1	0.59	.57	1.8	0.50	.48
T-C-A-T-T-C-G	1.9	1.9	1.01	.99	3.4	1.68	.59	0.7	0.41	.55
A-C-A-C-T-T-A	1.7	2.1	1.26	.82	2.3	1.05	.96	1.9	1.07	.95
Other	6.5	14.6	2.16	.46	11.3	1.81	.28	18.3	3.12	.09
Haplotype block 2 (stringent *r*^2^ threshold)										
A-C	80.5	73.1	0.66	.20	73.4	0.67	.26	72.7	0.64	.22
C-T	18.1	23.9	1.42	.30	24.9	1.50	.28	22.7	1.33	.45
Other	1.4	3.0	2.16	.46	1.7	1.21	.87	4.6	3.37	.06

#### 3.3.2 Treatment response analysis

When comparing the haplotype distribution between TRDI patients and MDNTR patients we did not find significant differences both with the stringent *r*^2^ threshold haplotype block and the lenient *r*^2^ threshold haplotype block (*P* = .17 and .15, respectively). The combinations of A-C and A-A-G-C-C-C-G were the dominant haplotypes both in the MDNTR patients and the TRDI patients. All results are shown in Table 5.

**Table 5:** Associations of haplotypes between *FKBP5* SNPs and treatment response.

Haplotype combination	MDNTR frequency %	TRDI frequency %	OR	*P*
Haplotype block 1 (lenient *r*^2^ threshold)				
A-A-G-C-C-C-G	61.3	58.5	0.85	.60
T-C-A-T-T-T-A	8.6	5.9	0.69	.53
A-A-A-C-C-T-G	3.7	5.4	1.47	.60
A-A-G-C-C-C-A	1.9	1.2	0.89	.92
T-C-A-T-T-T-G	4.2	5.2	1.19	.80
A-A-A-C-C-T-A	1.3	1.0	0.82	.89
A-A-A-C-C-C-G	2.1	1.8	1.24	.87
T-C-A-T-T-C-G	3.4	0.7	0.24	.36
A-C-A-C-T-T-A	2.3	1.9	0.72	.74
Other	11.3	18.3	1.77	.18
Haplotype block 2 (stringent *r*^2^ threshold)				
A-C	73.4	72.7	0.97	.92
C-T	24.9	22.7	0.89	.73
Other	1.7	4.6	2.78	.28

### 3.4 Power analysis

Given the sample size this study had limited power. Post-hoc power analyses showed that the power to detect the observed odds ratios for MDD cases versus healthy controls ranged from 0.192 to 0.645. For MDNTR cases versus healthy controls it was between 0.105 and 0.593 and for TRDI cases versus healthy controls between 0.113 and 0.752. The power to detect differences between TRDI and MDNTR cases ranged from 0.100 to 0.250.

## 4 Discussion

Candidate gene SNP association analysis is a commonly used method to delineate the role of genetic factors in the pathophysiology of MDD but also in pharmacogenomic approaches in predicting treatment response.^[34]^ In the present study we combined both approaches to investigate the role of *FKBP5* gene variants in MDD susceptibility and their usefulness as markers for predicting treatment response. We hypothesized that *FKBP5* polymorphisms could play an important role in increased MDD susceptibility and antidepressant treatment resistance, and in particular we wanted to test whether this association is more pronounced in TRDI patients. However, we did not find any significant difference in the distributions of alleles, genotypes, and haplotypes between cases and healthy controls or between TRDI patients and MDNTR patients, after correction for multiple testing.

Many GWAS for MDD have been carried out,^[35–38]^ but thus far little genome-wide significance has been reported,^[22,23,39]^ even though MDD has a strong genetic background with heritability estimates ranging from 31% to 42%.^[40]^ However, it also is known that MDD is a highly heterogeneous disorder. It is therefore important to note that in order to increase the sample size many GWAS have combined data from multiple sites. This will likely increase heterogeneity of the samples, which could partly explain the thus far disappointing results in MDD. In the present study the distribution of haplotype frequencies of rs1043805, rs3800373, rs9296158, rs7748266, rs1360780, rs9470080, and rs2817035 was significantly different between TRDI cases and healthy controls, but not between MDD cases and healthy controls or MDNTR cases and healthy controls. This might suggest a role of this haplotype block in the vulnerability for MDD, although significance did not survive multiple testing correction and no specific haplotype could be identified. The negative outcome when comparing the larger MDD sample with healthy controls might hint at a less homogeneous character of the combined sample. Furthermore, the comparison between TRDI patients and MDNTR patients for treatment response phenotype did not show any significance, which suggests that none of the investigated SNPs or haplotypes is involved in the resistance to antidepressant treatments.

*FKBP5* has been considered as a candidate gene for depression because of the role of the encoded protein in the HPA axis response to stress^[11]^ and its inhibition of inflammatory responses.^[16]^ First, recent studies have reported an association of *FKBP5* SNPs with treatment response in affective disorders. One study of 93 patients with bipolar disorder reported that SNPs rs1360789, rs9296158, and rs7748266, were associated with lithium response,^[41]^ whereas the TT genotype of rs1360789 was shown to be associated the response to antidepressant treatments in a STAR∗D cohort.^[42]^ Secondly, some variants of the *FKBP5* gene were also shown to be associated with the susceptibility for depression. For example, a cross-sectional study of 4639 samples reported that all minor alleles of rs9394309, rs9470080, rs7748266, and rs1360780 were not only associated with decreased levels of cortisol, but also with an increased likelihood of depressive symptoms.^[43]^ This circumstantial evidence suggests that *FKBP5* SNPs hold some promise as candidate markers for the pathophysiology of depression. In the present study, however, we could not demonstrate significant differences with any comparisons for SNPs and haplotype combinations of the *FKBP5* gene in relation to the depression and treatment response phenotypes, even in the homogeneous TRDI group. An important factor with our negative findings might relate to genetic differences between the Caucasians in the aforementioned studies^[19]^ and the Han Chinese in ours. This is corroborated by the CONVERGE project, which could not demonstrate a significant association of MDD with *FKNP5* gene in a homogenous population of Han Chinese.^[24]^ Furthermore, a study with an independent replication should be conducted to validate the findings.

The main limitation of our study is its relatively small sample size, although it was comparable with some recent MDD studies on treatment response.^[44,45]^ Power ranged only from 0.100 to 0.752. Enlarging the sample by including MDNTR patients and combining them with the TRDI patients may have negatively influenced the outcome, despite the increase in power, because of increased heterogeneity in the phenotype. Furthermore, the distribution of age and sex in the three groups are significantly different, which could affect the genetic association analysis even after correcting. Finally, genetic data were derived from 2 separately conducted studies both lacking detailed information necessary to adjust for confounding factors such as the antidepressants used, course of the disease and the number of episodes. It is clear that these confounding factors have no influence on the genetics, but they will influence the phenotype and thus potentially increase the heterogeneity of the samples.

In conclusion, we reported here, for the first time, that the *FKBP5* SNPs (rs1043805, rs3800373, rs9296158, rs7748266, rs136078,0 rs9470080, and rs2817035) and haplotypes were not associated with the susceptibility of MDD and treatment response to antidepressants in Han Chinese. There are many factors influencing MDD, such as presumable a high number of loci, their frequencies, their effect sizes, interactions with other genetic loci, but also environmental factors and interactions between them and genes. Without a proper understanding of the influence of these factors on MDD, the only practical way to improve the reliability of the results may be found in an increase of the sample size on the condition that homogeneity is not compromised.

### 4.1 Ethics approval and consent to participate

The 2 studies protocol were approved by the medical ethics committee of Tianjin Anding Hospital and conformed to “Declaration of Helsinki”. All participants had signed informed consent about the content and extent of the planned study before the participations. The patients’ guardians signed the informed consent on behalf of the participants when the capacity of participants to consent was compromised.

## Author contributions

Each author's individual contributions had been listed as following: Shen Li, Chenghao Yang, and Jie Li contributed to the conceptualization of this study; Fokko Bosker and Jie Li contributed to the planning; Yanyan Ma, Bing Chen, Meijuan Li, Chenghao Yang, and Shen Li conducted the implementation of experiments; Ilja Nolte worked out the data analysis; Chenghao Yang, Ilja Nolte, Fokko Bosker, and Jie Li contributed to the manuscript preparation.

**Conceptualization:** Chenghao Yang, Shen Li, Jie Li.

**Data curtain:** Ilja M. Nolte.

**Formal analysis:** Ilja M. Nolte.

**Funding acquisition:** Jie Li.

**Investigation:** Chenghao Yang, Shen Li, Yanyan Ma, Bing Chen, Meijuan Li.

**Supervision:** Jie Li.

**Writing – original draft:** Chenghao Yang, Fokko J. Bosker.

**Writing – review & editing:** Chenghao Yang, Fokko J. Bosker, Jie Li, Ilja M. Nolte.
